# Ultralow‐Power Peptide‐Based Memristor Enabled by Emulation of Proton‐Mediated Synaptic Signaling

**DOI:** 10.1002/smtd.202501472

**Published:** 2025-10-24

**Authors:** Jeong Hyun Yoon, Wooho Ham, Kyung Jun Park, Seok Daniel Namgung, Min‐Kyu Song, Jang‐Yeon Kwon

**Affiliations:** ^1^ School of Integrated Technology Yonsei University Incheon 21983 Republic of Korea; ^2^ BK21 Graduate Program in Intelligent Semiconductor Technology Yonsei University Incheon 21983 South Korea; ^3^ School of Electrical and Electronics Engineering Chung‐Ang University Seoul 06974 Republic of Korea; ^4^ School of Electrical Engineering Korea University 145 Anam‐ro, Seongbuk‐gu Seoul 02841 Republic of Korea

**Keywords:** bimodal memristor, memristor, neuromorphic computing, peptide material, proton‐mediated signaling

## Abstract

The substantial power consumption of traditional computing architectures, arising from the physical separation of memory and processing units, has motivated the exploration of neuromorphic systems that emulate the remarkable energy efficiency of the human brain. However, artificial neural networks implemented on neuromorphic devices still require billions of weight updates, and conventional devices typically consume power in the milliwatt range per switching event, posing a significant challenge even for neuromorphic systems. In this study, a synapse‐like memristive device utilizing a tyrosine‐rich peptide is presented as the resistive switching layer. By emulating proton‐mediated signaling of biological synapses, the device leverages proton–electron dual‐carrier transport enabled by the redox‐active properties of tyrosine to realize ultralow‐power resistive switching. Proton modulation is implemented through two methods: i) exposure to external humidity and ii) electrically driven injection using a PdH_x_ proton reservoir layer. Comparative analysis reveals that the electrically driven approach achieves an ultralow switching power of 215 pW—≈2500 times lower than that of the intrinsic device—primarily owing to the sustained low off‐current during proton injection. These results demonstrate a promising strategy for developing highly energy‐efficient, synapse‐like memristive devices through precise control of proton dynamics.

## Graphical abstract

A tyrosine‐rich peptide‐based memristor emulates proton‐mediated synaptic signaling through dual protonelectron conduction. Proton modulation via humidity or PdHx injection enables ultralow‐power switching (215 pW), ≈2500× lower than intrinsic devices. This work demonstrates a biorealistic, energy‐efficient strategy for neuromorphic electronics through precise control of proton dynamics and redox coupling.

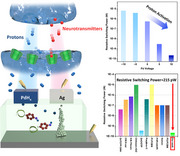

## 1 Introduction

The era of artificial intelligence (AI) has arrived, with applications rapidly expanding across diverse fields such as autonomous driving, finance, and healthcare.^[^ ^1^, ^2^, ^3^ ^]^ Consequently, the market value of AI technologies continues to increase exponentially.^[^ ^4^, ^5^, ^6^ ^]^ However, conventional von Neumann architectures suffer from inefficiencies due to the physical separation of memory and processing units, leading to significant time and energy consumption during data transfer.^[^ ^7^, ^8^ ^]^ In contrast, the human brain, composed of ≈86 billion neurons and 100 trillion synapses, can store up to 2.5 petabytes of data and perform operations at a petaflop scale, all while consuming only ≈20 watts of power.^[^ ^9^, ^10^, ^11^, ^12^ ^]^ Inspired by this unparalleled efficiency, extensive research is being conducted to develop brain‐like processors with high degrees of parallelism, aiming to overcome the limitations of traditional computing systems.^[^ ^13^, ^14^, ^15^, ^16^, ^17^, ^18^ ^]^

Memristors, one of the most promising candidates for next‐generation neuromorphic hardware owing to their high integration density and scalability, can closely resemble biological synapses owing to its simple two‐terminal metal—insulator—metal (MIM) structure, and perform synapse‐like computation.^[^ ^15^, ^16^, ^17^, ^19^, ^20^, ^21^ ^]^ However, memristor‐based artificial neural networks (ANNs) require up to 10 billion weight updates, even for relatively simple datasets such as MNIST (28 × 28 pixel handwritten images).^[^ ^22^, ^23^ ^]^ Since conventional memristive devices typically consume power on the order of milliwatts per switching, the memristor with lower resistive switching power is essential for practical neuromorphic implementation.^[^ ^24^, ^25^ ^]^

In biological synapses, proton release through ion channels forms an extracellular pH gradient—‐a process known as proton‐mediated signaling—which is fundamental to neurotransmission.^[^ ^26^, ^27^, ^28^, ^29^ ^]^ This mechanism provides the driving force required for neurotransmitter storage and release.^[^ ^30^, ^31^, ^32^ ^]^ Moreover, the reuptake of key neurotransmitters such as monoamines, GABA, and glutamate depends on the H⁺ gradient, underscoring the critical role of proton dynamics in regulating neurotransmission efficiency.^[^ ^33^, ^34^ ^]^

To emulate this biological mechanism, we developed a memristor driven by dual carriers—protons and electrons. Specifically, inspired by natural proton‐coupled electron transfer systems, we fabricated a memristor using a tyrosine‐rich peptide (YYACAYY, Y7C) as the resistive switching layer.^[^ ^23^, ^35^, ^36^ ^]^ The peptide layer exhibited proton‐mediated switching behavior, with the set voltage decreasing under higher humidity. This behavior is attributed to the unique properties of tyrosine, which promote the spontaneous formation of a two‐dimensional helical dimer film and, when hybridized with manganese oxide, exhibit enhanced conductivity.^[^ ^37^, ^38^ ^]^ However, to truly replicate the energy efficiency of proton‐mediated synaptic signaling, low set voltages must also be accompanied by low off‐currents, as switching power is defined as PSET  =  VSET  ×  ISET.^[^ ^39^, ^40^ ^]^ While higher humidity reduces the set voltage, it also substantially increases the off‐current, thereby negating the intended low‐power operations.

To overcome this limitation, we propose a novel strategy: direct proton injection into the peptide layer. This approach reduces the operating voltage of the memristor while maintaining low off‐current characteristics. Electrochemical impedance spectroscopy (EIS) confirmed that the resistivity of the Y7C peptide decreases with increasing humidity, consistent with its tyrosine‐rich structure. To replace humidity‐driven modulation with electrical control, palladium electrodes—well known for their proton permeability—were integrated on top of the peptide layer. Controlled proton injection was verified by analyzing transient currents between the palladium electrodes. Furthermore, a three‐terminal device was fabricated in which an external palladium electrode, exposed to a hydrogen‐rich environment, served as a proton reservoir. By modulating the voltage applied to this terminal from −10 to +10 V, we achieved a 6.9‐fold variation in the set voltage. Remarkably, this configuration yielded an ultralow switching power of only 215 pW—≈2500 times lower than that of the intrinsic peptide memristor in ambient air. This dramatic reduction in switching energy is attributed to the significantly lower off‐current enabled by electrically injected protons, which enhanced the conductivity of protonated peptide films.

## 2 Results

### 2.1 Proton‐Mediated Resistive Switching

**Figure 1:**
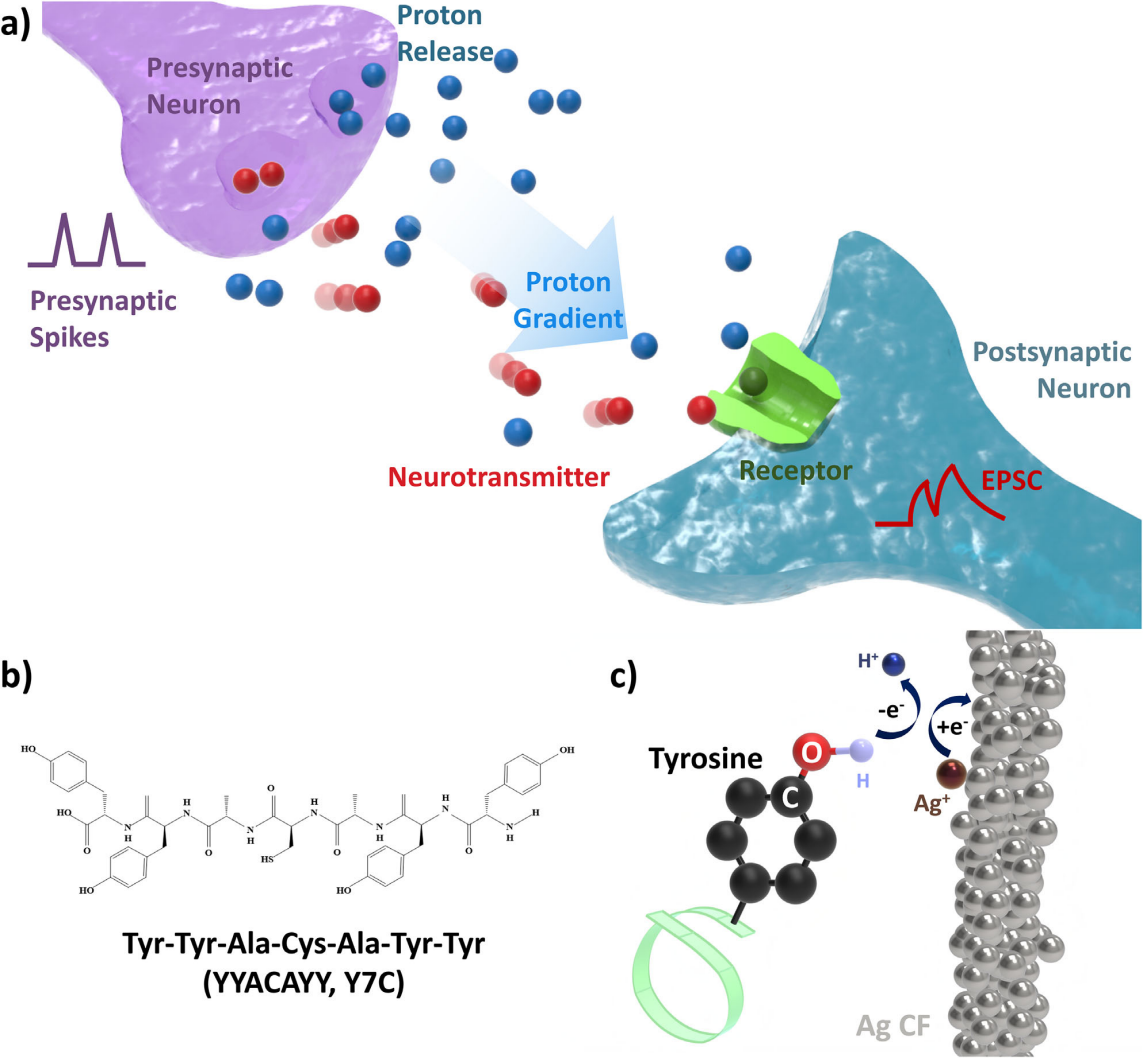
Proton‐mediated signaling in biological synapses and the peptide material with proton–electron dual‐carrier conduction. a) Schematic of proton‐mediated signaling in a biological synapse. Protons are co‐released with neurotransmitters from synaptic vesicles, producing transient extracellular acidification that can modulate synaptic signaling. b) Molecular structure of the Y7C peptide. The phenolic groups of tyrosine and thiol functionalities of cysteine are highlighted in red and blue, respectively. c) Electron transfer between tyrosine residues and silver ions. During tyrosine deprotonation, silver cations are reduced to neutral silver atoms. Each deprotonation involves the donation of one electron and one proton by tyrosine.

Proton‐mediated signaling is a biological process in which protons are co‐released with classical neurotransmitters at the synapse, while the vesicular proton gradient generated by V‐ATPase provides the driving force for energy‐efficient neurotransmitter loading into synaptic vesicles^[^ ^26^, ^27^, ^28^ ^]^ (**Figure** **1**a). Furthermore, in the central nervous system, the acid‐sensing ion channels function as specific proton sensors, responding to extracellular pH fluctuations within the physiologically relevant range of ≈5–8. Inspired by this energy‐efficient biological process, we implemented dual‐carrier conduction—protons and electrons—within a memristor device.

To this end, we synthesized a peptide with the sequence YYACAYY (Y7C) and confirmed its ability to form a stable thin film through helix dimerization, facilitated by disulfide bonding between the cysteine residues (Figure 1b; Figure S1, Supporting Information).^[^ ^36^, ^37^ ^]^ Owing to the redox‐active properties of tyrosine and the low oxidation potential of the Y7C peptide, consecutive proton‐coupled redox reactions between tyrosine and Ag ions facilitate the efficient transport of Ag clusters and conductive filaments formation.^[^ ^27^, ^41^, ^42^, ^43^, ^44^ ^]^ Under high‐humidity conditions, we observed a significant reduction in the set voltage required to form conductive filaments. This behavior is attributed to consecutive redox reactions between Ag⁺ cations and phenolic hydroxyl groups, which facilitate energy‐efficient filament formation (Figure 1c).^[^ ^35^, ^36^, ^45^ ^]^

### 2.2 Proton Modulation Approaches

**Figure 2:**
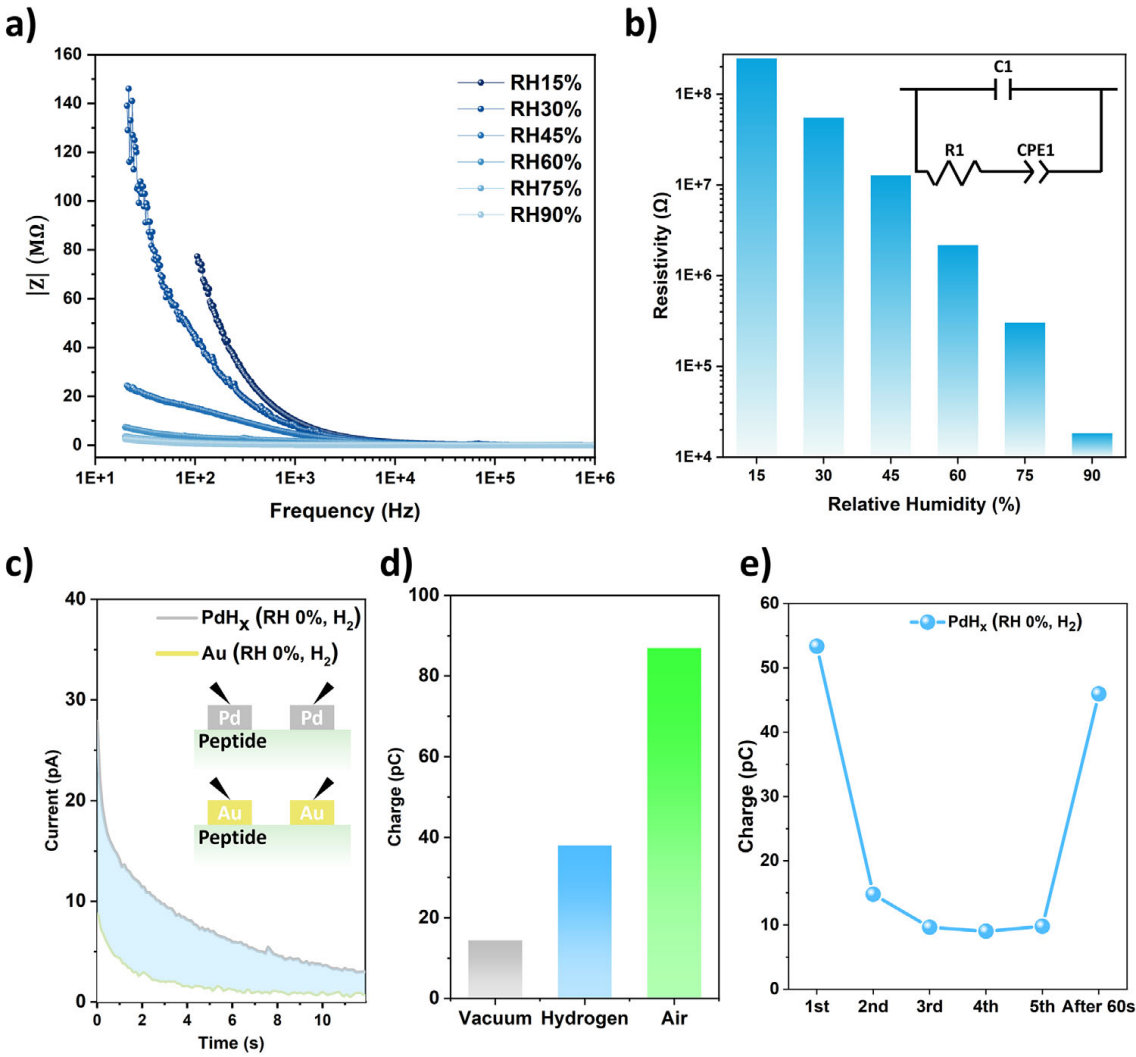
Proton injection through the peptide layer via external humidity and PdH_x_ contacts. a) Impedance magnitude of Y7C peptide films measured across 20–10⁶ Hz at RH levels from 15% to 90%. b) Proton resistivity derived from impedance data at each RH level. c) Transient current measured between two Pd/Au contacts under a hydrogen atmosphere at RH 0%.d) Total proton charge transferred between two Pd electrodes under vacuum (gray), hydrogen (blue), and ambient air at RH 50% (green). (e) Proton charge recovery after five consecutive transient current measurements under hydrogen (RH 0%), followed by 60 s of rest. Charge values were calculated by integrating transient currents over time.

To investigate the proton injection effects on memristor switching voltage, both humidity‐based and electrical inputs were applied, and the resulting changes within the peptide film were analyzed. The proton resistivity of Y7C peptide thin films was evaluated using EIS. Proton‐blocking Au electrodes were used as top contacts to isolate and estimate the proton‐specific resistivity.^[^ ^46^, ^47^ ^]^ Impedance measurements were conducted over the frequency range of 100–10^6^ Hz. The magnitude of the impedance as a function of frequency is presented in **Figure** **2**a. The proton resistivity was quantitatively extracted by fitting the impedance data to a resistor‐capacitor (RC) equivalent circuit model, which is commonly used in proton exchange membrane analysis (Figure 2b).^[^ ^48^, ^49^, ^50^ ^]^ As relative humidity (RH) increased, a sharp decrease in proton resistivity was observed from 2.5 × 10^8^ Ω at 15% RH to 1.85 × 10^4^ Ω at 90% RH, corresponding to a 13 500‐fold reduction.

To modulate the resistive switching characteristics through RH control, protons were injected into the memristor device using an electrical input. To evaluate the feasibility of this approach, two Pd electrodes—known for their proton permeability^[^ ^38^, ^51^ ^]^—were deposited with a 100 µm gap on the Y7C peptide layer. A voltage of 10 V was applied to one electrode while the other electrode was grounded, and the time‐dependent current response was measured under three environmental conditions: dry vacuum (RH 0%), hydrogen atmosphere (5% H_2_ in Ar, RH 0%), and ambient humid air (RH 50%). Identical devices with two Au electrodes serving as proton‐blocking contacts were fabricated and tested under the same conditions (Figure 2c).^[^ ^46^, ^47^ ^]^ Pd absorbs and conducts hydrogen from the environment, whereas Au serves as a barrier against ionic conduction. The amount of proton conduction was estimated by calculating the charge difference between the PdH_x_ and Au devices under identical conditions. The estimated proton conduction was 14.4 pC in dry vacuum, 38 pC in a hydrogen atmosphere at RH 0%, and 87 pC in ambient air at RH 50% (Figure 2d).

In addition, the transient current measured under hydrogen and humid air conditions exhibited distinct recharge behaviors after 60 s of exposure to ambient air, following several consecutive measurements. In the Pd–Pd structured device, the transient current under a hydrogen atmosphere showed a decreasing trend with repeated pulses. However, after a 60 s recovery period, the current returned to 91% of its initial value, indicating effective proton recharging under a hydrogen atmosphere, which conducted between Ag and PdH_x_ electrodes, across the 100 µm gap. (Figure 2e; Figure S2, Supporting Information).^[^ ^48^, ^52^, ^53^ ^]^ In contrast, under humid air, the current also decreased during consecutive measurements, but recovered to less than 60% of the initial value after reset (Figure S3, Supporting Information), suggesting that transient currents in humid conditions were not primarily due to proton conduction into the peptide film. Unlike hydrogen, which enabled effective and reversible proton injection via PdH_x_, humid air provided limited and less reversible proton transport.

### 2.3 Resistive Switching Power Reduction Through Proton Injection

To evaluate the effect of proton injection on the switching power of peptide‐based memristor devices exhibiting proton–electron dual‐carrier resistive switching behavior, two types of devices were fabricated:^[^ ^23^, ^36^ ^]^

**Figure 3:**
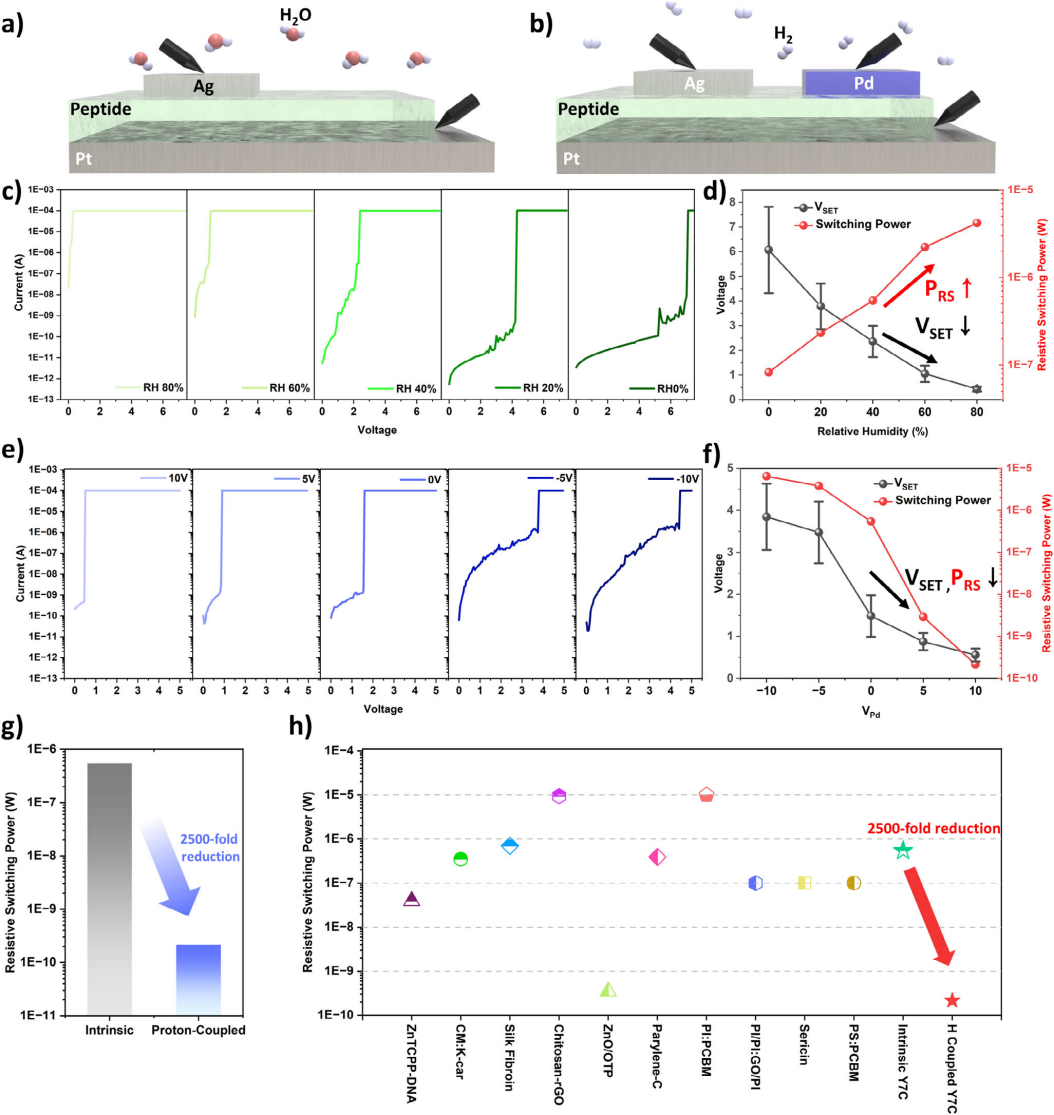
Variation of V_SET_ and P_RS_ under external humidity and V_Pd_. a,b) Schematics of two peptide‐based memristors with reduced set voltages via proton injection: (a) through external humidity, and (b) via voltage applied to an adjacent proton‐permeable PdH_x_ electrode in a hydrogen atmosphere. c) Representative *I–V* curves of the peptide memristor under RH levels ranging from 0% to 80% in 20% increments. d) Average set voltage and switching energy at each RH level. e) *I–V* curves of the memristor under 45% RH hydrogen atmosphere as a function of the voltage applied to the adjacent PdH_x_ electrode. f) Corresponding average set voltage and switching energy for each applied V_Pd_. df) The set voltages are presented as the mean ± standard deviation (n = 30). (d) In the RH mode, the SD% values (standard deviation divided by the mean) were ≈28.9%, 24.4%, 26.9%, 31.7%, and 9.6%, in order of increasing relative humidity. (f) In the Pd mode, the corresponding SD% values were 20.5%, 21.2%, 33.2%, 23.3%, and 27.5%, respectively, in order of increasing applied V_Pd_. g–h) Comparison of resistive switching power consumption for (g) the intrinsic peptide memristor (grey) vsthe proton‐injected dual‐carrier‐coupled peptide memristor (blue), and (h) previously reported memristor devices based on organic switching media. [Correction added on November 1, 2025, after first online publication: Figure 3 has been updated.]

1. A conventional Ag/Y7C/Pt structure in which resistive switching is modulated by RH.
2. A modified device incorporating a Pd‐based proton insertion layer located 100 µm adjacent to the memristor's active region, enabling electrical control of proton injection (**Figure** **3**a,b; Figure S4, Supporting Information).

In the Ag/Y7C/Pt device, as shown in Figure 3c and Figure S5a (Supporting Information), increasing RH from 0% to 80% led to a substantial decrease in the set voltage from 6.07 ± 1.75 to 0.43 ± 0.096 V, suggesting favorable conditions for low‐power operation. However, as the switching power (P_set_ = I_set_ × V_set_) is closely related to the off‐state current, an increase in RH, despite lowering the set voltage, may result in higher overall switching power if the current in the high‐resistance state (HRS) also increases. Specifically, the device exhibited an off‐current (V_Read_ = 0.1 V) of 4.5  ×  10^−12^ A under 0% RH, which increased drastically to 1.5 × 10^−6^ A under 80% RH. Also, cycle endurance characteristics also showed humidity‐varying HRS resistance values (from 10^12^Ω at RH 0% to 10^7^Ω at RH 80%, Figure S6, Supporting Information). Consequently, the power required for resistive switching increased from 82.2 nW at 0% RH to 4.19 µW at 80% RH, despite the decrease in set voltage. This inverse relationship is primarily attributed to the significant increase in off‐state current under higher humidity conditions (Figure 3d).

To further explore the influence of RH on the resistive switching characteristics (set voltage and off‐current), a kinetic isotope effect (KIE) study was conducted by introducing D_2_O vapor instead of H_2_O vapor. The set voltages were measured at RH levels ranging from 15% to 75% in 15% increments. With D_2_O vapor, the set voltage decreased from 5.71 to 0.86 V, following a trend similar to that observed with H_2_O vapor. However, the average set voltage in D_2_O was ≈62.2% higher than that in H_2_O. Notably, at RH levels of 30%, 45%, and 60%, the device consistently required ≈73% higher voltage under D_2_O vapor to achieve memristor set operation (Figure S7, Supporting Information). Considering that the set voltage in a memristor represents the electrical bias required for the formation of conductive filaments, the higher set voltage observed under D_2_O vapor conditions indicates that the presence of heavier deuterium imposes greater electrical stress on the complex process involving silver redox reactions and ionic conduction. This kinetic isotope effect provides compelling evidence that hydrogen species present in ambient humidity are incorporated into the peptide layer as protons, actively contributing to the modulation of the resistive switching behavior of the device.^[^ ^36^ ^]^

By contrast, the three‐terminal memristor device with a Pd proton insertion layer demonstrated voltage‐controlled modulation of switching behavior^[^ ^23^ ^]^ Applying a positive voltage (V_Pd_ = + 10 V) to the Pd electrode resulted in proton injection into the memristor region, facilitating Ag atom migration through redox‐active tyrosine residues and enabling conductive filament (CF) formation at a significantly reduced set voltage of 0.56 ± 0.15 V. Conversely, applying a negative voltage to the Pd electrode depleted protons from the active region, increasing the energy barrier for CF formation and resulting in a higher set voltage of 3.84 ± 0.79 V (Figure 3e; Figure S5b, Supporting Information).

Unlike the RH‐based modulation method, the three‐terminal device maintained a consistent off‐current of ≈2  ×  10^−10^ A, regardless of the applied bias to the PdH_x_ proton reservoir layer. This stability also resulted in a consistent HRS resistance over 30 consecutive switching cycles (Figure S8, Supporting Information). Consequently, the power required for resistive switching decreased with increasing positive bias to the Pd terminal, reaching a minimum of 2.15  ×  10^−10^ W at V_Pd_ = +10 V. This indicates that the resistive switching power of a proton–electron dual‐carrier memristor can vary by up to ≈30 000‐fold depending on proton modulation through PdH_x_ proton reservoir layer (Figure 3f). Furthermore, by increasing the local proton concentration in the filament formation region via the proton reservoir layer, the P_RS_ value was reduced by ≈2,500‐fold compared with that of the intrinsic peptide memristor operated under ambient air conditions (RH 40%) (Figure 3g). Compared to ten previously reported biomaterial‐based memristor devices, and even inorganic material‐based memristor devices, the intrinsic peptide memristor exhibited a P_RS_ value close to the median, whereas the device with proton‐coupled resistive switching achieved the lowest P_RS_ value^[^ ^54^, ^55^, ^56^, ^57^, ^58^, ^59^, ^60^, ^61^, ^62^, ^63^, ^64^, ^65^, ^66^, ^67^, ^68^, ^69^, ^70^ ^]^ (Figure 3h; Figure S9, Supporting Information).

To further investigate the proton injection process, two additional verification experiments were conducted. First, to exclude humidity effects, the set voltage of the memristor was measured as a function of the applied Pd electrode bias (V_Pd_) under a hydrogen atmosphere at 0% RH and under vacuum. As expected, the overall VSET values were higher at 0% RH than at 45% RH because of the increased resistivity at low humidity (Figure 2b). Nonetheless, a clear decrease in V_SET_ with increasing V_Pd_ was observed under the hydrogen atmosphere, whereas this effect disappeared under vacuum (Figure S10, Supporting Information). This indicates that protons originating from ambient hydrogen were injected along the Pd electrode, thereby lowering the electrical threshold for conductive filament formation. Second, to directly confirm proton injection through the Pd electrode, the electrode spacing between the Ag and Pd contacts was increased from 100 to 500 µm and 1000 µm. In these devices, the set voltage became invariant with respect to V_Pd_, confirming that switching‐voltage modulation occurs only under a hydrogen atmosphere and is directly attributable to proton injection via the Pd electrode (Figure S11, Supporting Information).

### 2.4 Resistive Switching Under Two Proton Modulation Modes: RH and Reservoir Layer

**Figure 4:**
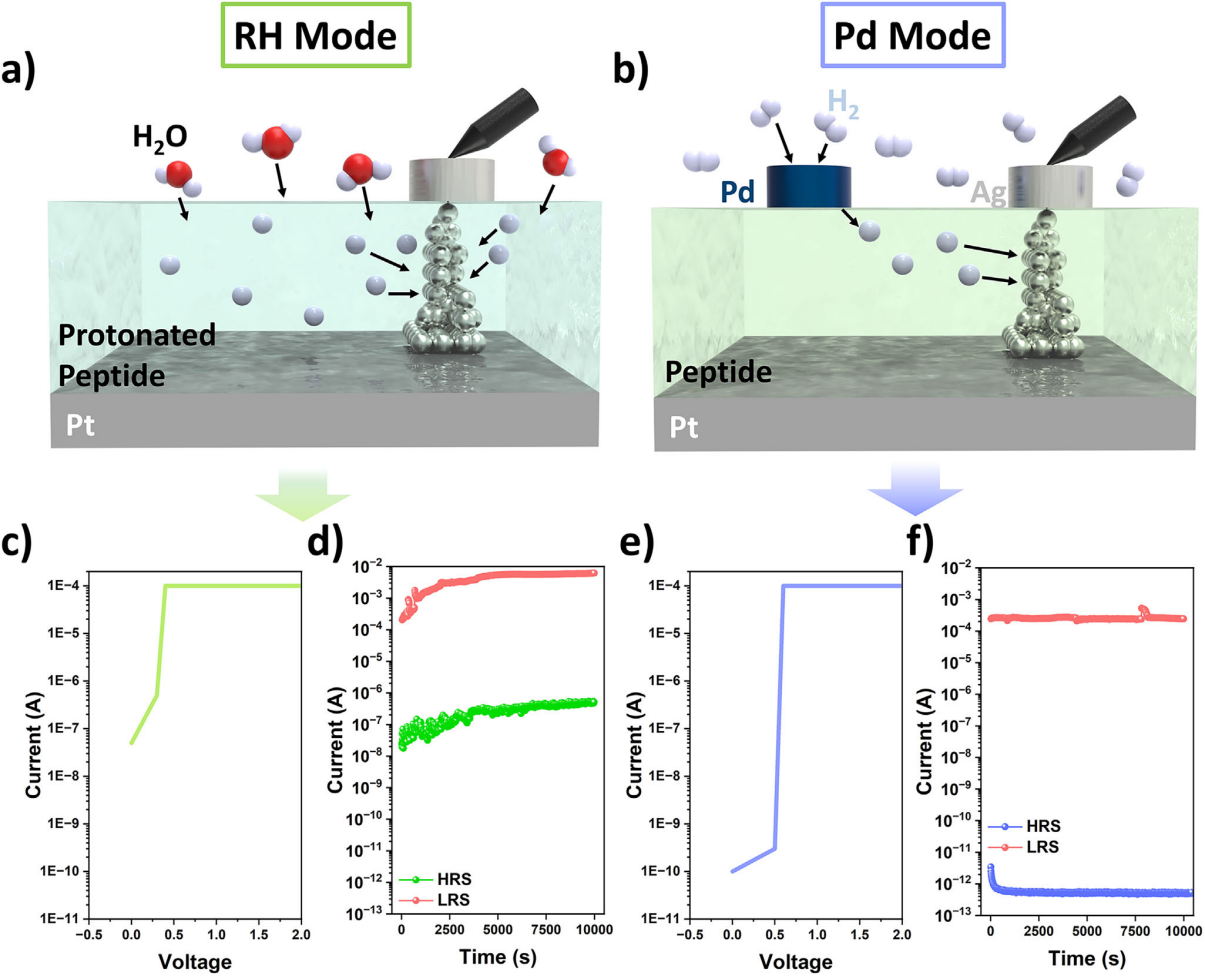
Proton injection mechanisms and corresponding conductive filament formation in two modulation modes (RH and Pd). a,b) Schematics of filament formation induced by proton injection via (a) external humidity and (b) a PdH_x_ contact. c,d) RH mode: (c) *I–V* curve and (d) 10^4^ s retention characteristics at 80% RH, the condition yielding the lowest set voltage. e,f) Pd mode: (e) *I–V* curve and (f) 10^4^ s retention characteristics under a +10 V bias applied to the PdH_x_ electrode, the condition producing the lowest set voltage.

As illustrated in Figure 3d–f, although both RH and the application of voltage to a proton reservoir layer (PdH_x_) effectively reduced the set voltage by facilitating proton incorporation, the impact on switching energy varied significantly between the two methods. In the RH‐controlled device, protons from H_2_O vapor were directly introduced onto the surface of the peptide layer.^[^ ^35^, ^36^ ^]^ The number of protons injected using this method was greater than that in the Pd mode, where protons were introduced by applying a voltage to the adjacent Pd electrode in a hydrogen atmosphere (Figure 2d). However, this increased proton incorporation was accompanied by a marked decrease in the resistivity of the Y7C peptide thin film from 1.27 × 10⁷ Ω under dry conditions to below 3 × 10⁵ Ω at high RH, representing more than a 100‐fold reduction (**Figure** **4**a). Consequently, the off‐current at V_Read_ = 0.1 V increased dramatically, from hundreds of picoamperes to several hundred nanoamperes—an increase of more than three orders of magnitude (Figure 4c). This substantial leakage current ultimately led to higher energy consumption during resistive switching under humid conditions.

By contrast, the three‐terminal device incorporating a proton reservoir layer allowed protons to be selectively driven into or extracted from the memristor region via an applied electric field, depending on the polarity of the voltage applied to the PdH_x_ electrodes (Figure 4b)^[^ ^23^ ^]^ This electrically controlled injection modulated proton availability within the conductive filament‐forming region without altering the intrinsic resistivity of the peptide thin film. Consequently, the off‐current remained consistently low (≈ 100pA), regardless of the bias applied to the proton reservoir electrodes (Figure 4e). Both device configurations maintained their respective resistive states for 10^4^s. However, under high RH conditions (80%), the increased intrinsic conductivity of the peptide film (Figure 2a,b) limited the on/off current ratio to ≈10⁴ in the RH‐based mode. In contrast, the Pd‐driven mode achieved a significantly higher on/off ratio exceeding 10⁸ (Figure 4d–f; Figures S12 and S13, Supporting Information).

## 3 Conclusion

In summary, we demonstrated proton injection into a memristor incorporating a tyrosine‐rich peptide layer using two distinct methods: external humidity and electrically driven proton injection. Both approaches significantly reduced the set voltage, with electrically controlled injection via a PdH_x_ contact achieving an 11.6‐fold reduction in switching power. This result successfully emulates proton‐mediated signaling, the key mechanism enabling energy‐efficient neurotransmission in biological synapses. Although the present implementation requires a hydrogen atmosphere, integration with solid‐state proton reservoirs could enable scalable ultralow‐power neuromorphic devices. By emulating the complex biochemical processes that underlie the energy efficiency of the human nervous system, this study advances the development of brain‐inspired synaptic devices beyond conventional structural limitations.

## 4 Experimental Section

### Device Fabrication and Characterization

A 10‐nm Cr adhesion layer, followed by a 100‐nm Pt bottom electrode, was deposited onto SiO_2_/Si substrates via direct current (DC) sputtering. The peptide thin film was prepared by dissolving peptide powder (Scipeptide, 97% purity) in trifluoroacetic acid (TFA; Daejung, 99%) and spin‐coating the resulting solution onto the Cr/Pt‐coated substrates at 4000 rpm (Figure S1, Supporting Information). Circular Ag and Pd top electrodes, each 200‐nm thick, were deposited on the Y7C peptide film using electron‐beam evaporation at a deposition rate of 2.0 nm s^−1^ through shadow masks with a 100‐µm radius.

Impedance spectroscopy of the peptide layer was conducted using a Keysight E4990A impedance analyzer over a frequency range of 20 Hz–1 MHz. The real and imaginary components of the impedance, along with magnitude and phase, were recorded. Measurements were performed using a 500‐mV oscillator signal and a 100‐mV DC bias.

For transient current analysis, a test configuration was fabricated by depositing two identical Pd/Au electrodes, separated by a 100‐µm gap, onto the spin‐coated Y7C film. A constant voltage was applied to one electrode, and the current response was measured at the other. Humidity within the test chamber was controlled using humidified air and monitored in real time with a Sensirion SHT31 humidity sensor. All electrical measurements were conducted using a Keithley 4200‐SCS parameter analyzer.

### Statistical Analysis

In Figure 3d,f, the set voltages of the RH mode and Pd mode devices were plotted as a function of relative humidity (RH) and applied Pd electrode voltage (V_Pd_), respectively. Each error bar represents the mean ± standard deviation (SD) calculated from 30 individual measurements. In Figure S7 (Supporting Information), the set voltage of the Y7C peptide‐based memristor was presented as a function of relative humidity under both H_2_O and D_2_O vapor environments. The dotted lines indicate linear fits obtained using the Origin software, yielding R^2^ values of 0.973 for D_2_O and 0.915 for H_2_O, respectively. In Figure S10 (Supporting Information), the set voltages of the Pd mode Y7C memristor under dry hydrogen atmosphere (RH 0%) and vacuum conditions were displayed with error bars representing mean ± SD, based on 20 independent measurements for each condition. In Figure S11 (Supporting Information), the set voltages of devices with increased electrode spacing (from 100 to 500 µm and 1000 µm between the Pd electrode and the memristor region) are plotted. For each V_Pd_ bias condition, the data points represent the average values calculated from 20 measurements.

## Conflict of Interest

The authors declare no conflict of interest.

## Supporting information

Supporting Information

## Data Availability

The data that support the findings of this study are available from the corresponding author upon reasonable request.
