# Supplementary material for: Ultralow‐Power Peptide‐Based Memristor Enabled by Emulation of Proton‐Mediated Synaptic Signaling
Source: Small Methods. 2025 Oct 24;9(12):e01472. doi: 10.1002/smtd.202501472 (PMC12716227; doi:10.1002/smtd.202501472)
Supplement: Supplementary file 1 — Supporting Information [file SMTD-9-e01472-s001.docx]

Supporting Informations:

**Ultralow-Power Peptide-Based Memristor Enabled by Emulation of Proton-Mediated Synaptic Signaling**

*Jeong Hyun Yoon^a,b^, Wooho Ham^a,b^, Kyung Jun Park^a,b^, Seok Daniel Namgung^c^,*

*Min-Kyu Song^d^*, Jang-Yeon Kwon^a,b^**

^a^ School of Integrated Technology, Yonsei University, Incheon 21983, Republic of Korea

^b^ BK21 Graduate Program in Intelligent Semiconductor Technology, Yonsei University, Incheon 21983, South Korea

^c^ School of Electrical and Electronics Engineering, Chung-Ang University, Seoul 06974, Republic of Korea

^d^ School of Electrical Engineering, Korea University, 145 Anam-ro, Seongbuk-gu, Seoul 02841, Republic of Korea

Keywords: memristor, neuromorphic computing, peptide material, proton-mediated signaling, bimodal memristor

**^*^ Corresponding authors:**

Min-Kyu Song, Ph.D.

School of Electrical Engineering, Korea University, Seoul 02841, Republic of Korea

Tel: +82-02-3290-3238

E-mail: mksong@korea.ac.kr

Jang-Yeon Kwon, Ph.D.
School of Integrated Technology, Yonsei University, Incheon 21983, Republic of Korea
Tel: +82-032-749-5837
E-mail: jangyeon@yonsei.ac.kr

**
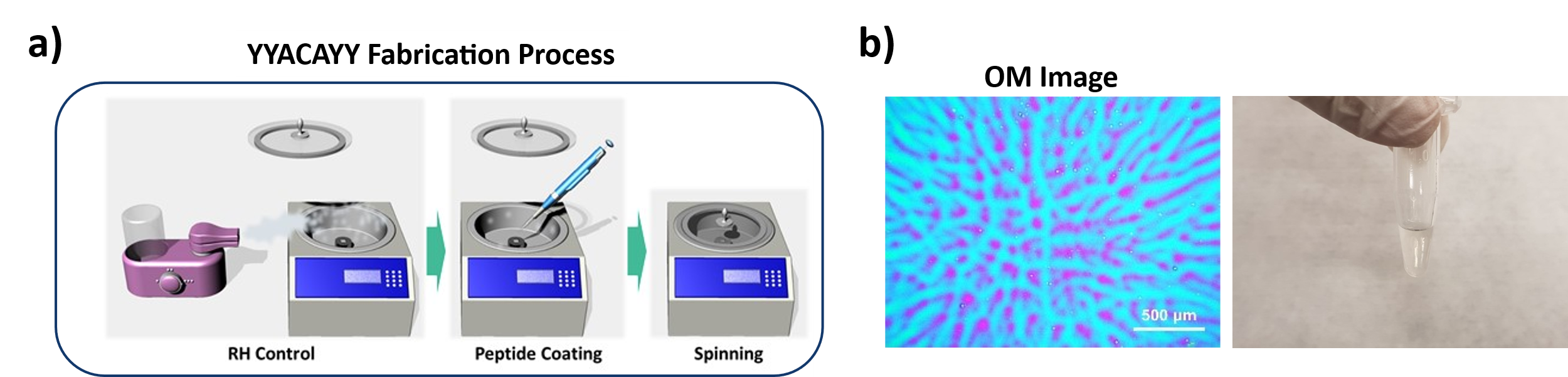
**

**Figure S1. (a)** Fabrication process of Y7C layer through spin coating and **(b)** optical microscope lateral image of Y7C film (left) and Y7C solution dissolved in trifluoroacetic acid (TFA) 99%. **
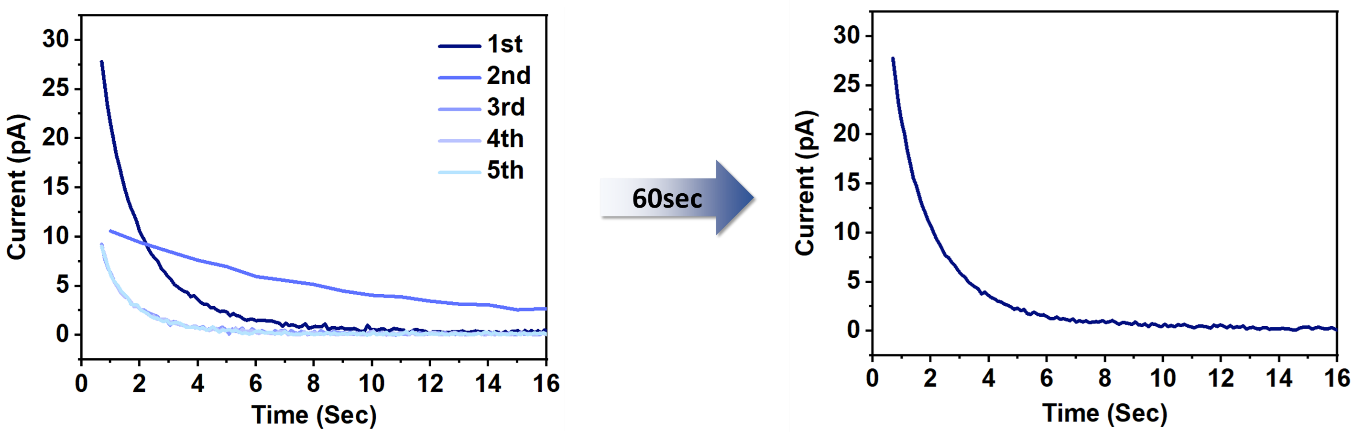
**

**Figure S2.** The transient current between 100 $\boldsymbol{\mu}$m gap Pd electrodes deposited on Y7C peptide layer was measured five consecutive times. After the 5^th^ transient current measurement, Pd contacts were exposed to RH 0% hydrogen atmosphere for 60 second and measured again.

**
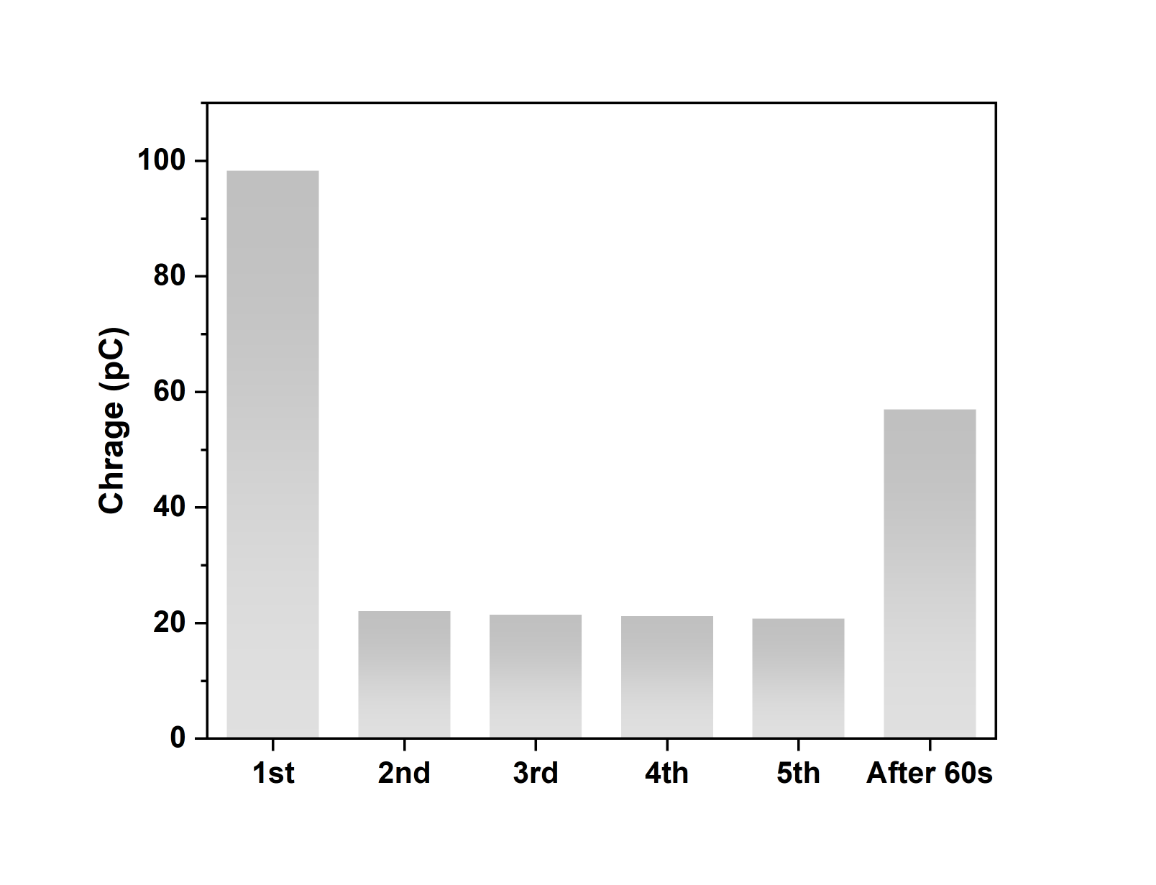
**

**Figure S3.** Total charge transferred by protons between two Pd electrodes on the Y7C peptide layer under ambient air with 50% relative humidity (RH). Following five consecutive transient current measurements, charge recovery was evaluated after 60 seconds of exposure to humid air. The transferred charge was estimated by integrating the transient current over time.

**
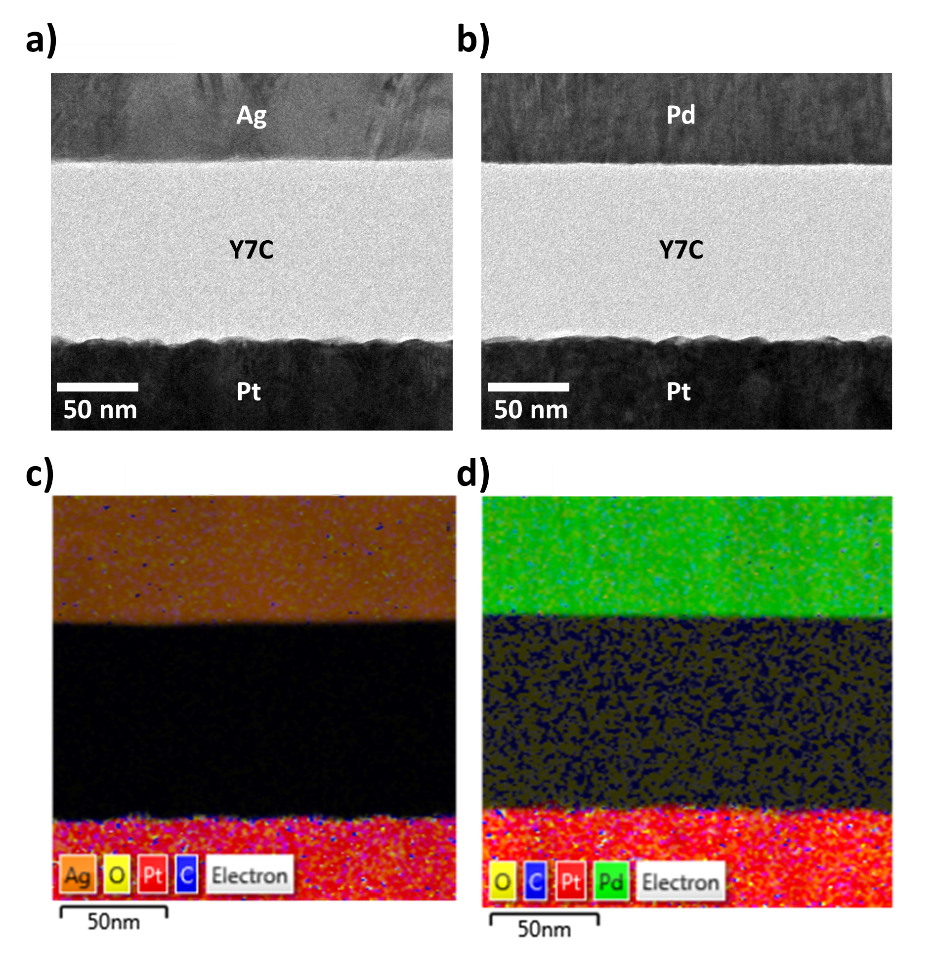
**

**Figure S4**. Transmission electron microscopy (TEM) and energy dispersive spectrometer (EDS) image of the Y7C film with Ag, Pd contacts. Through the high-resolution TEM, cross-section image of the Y7C film is obtained, with verification of 103nm thickness. Also, in the EDS image, Ag, Pt, C, and Pd are plotted in orange, red, blue, and green colors, respectively.**
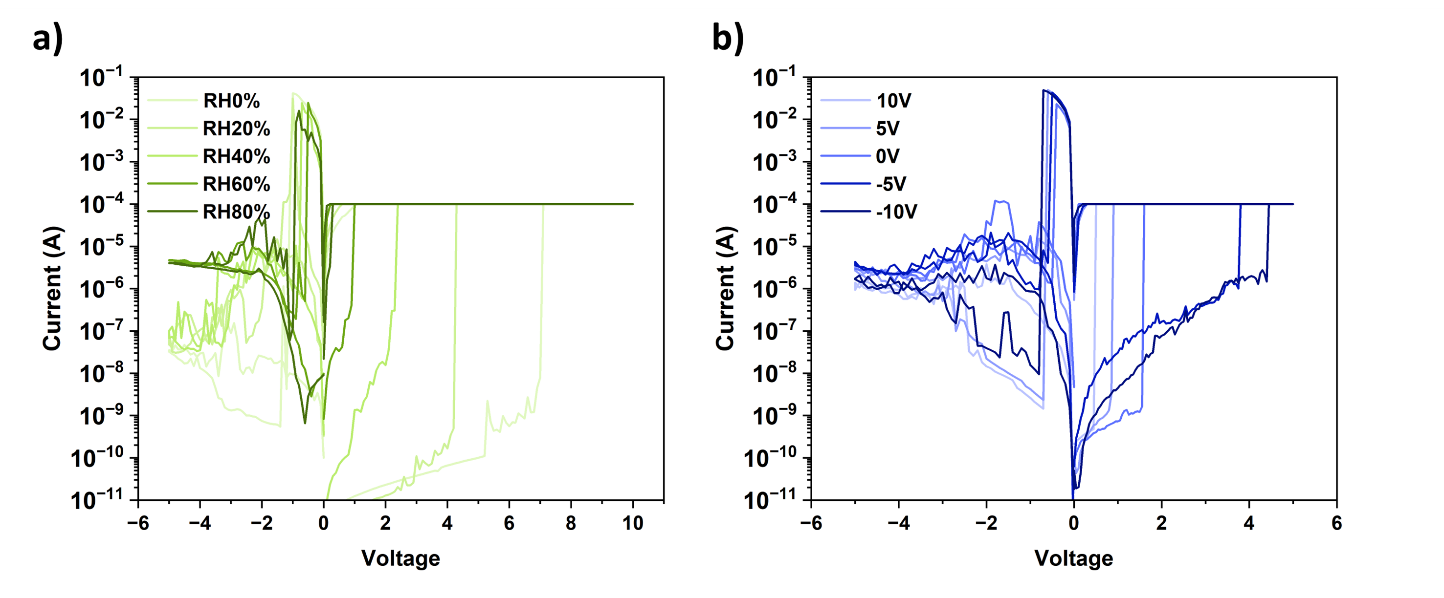
**

Figure S5. The set voltages of the Y7C peptide memristor as a function of the RH of H_2_O (blue) and D_2_O (green). Dotted lines indicate linear fitting of the experimental results.

**
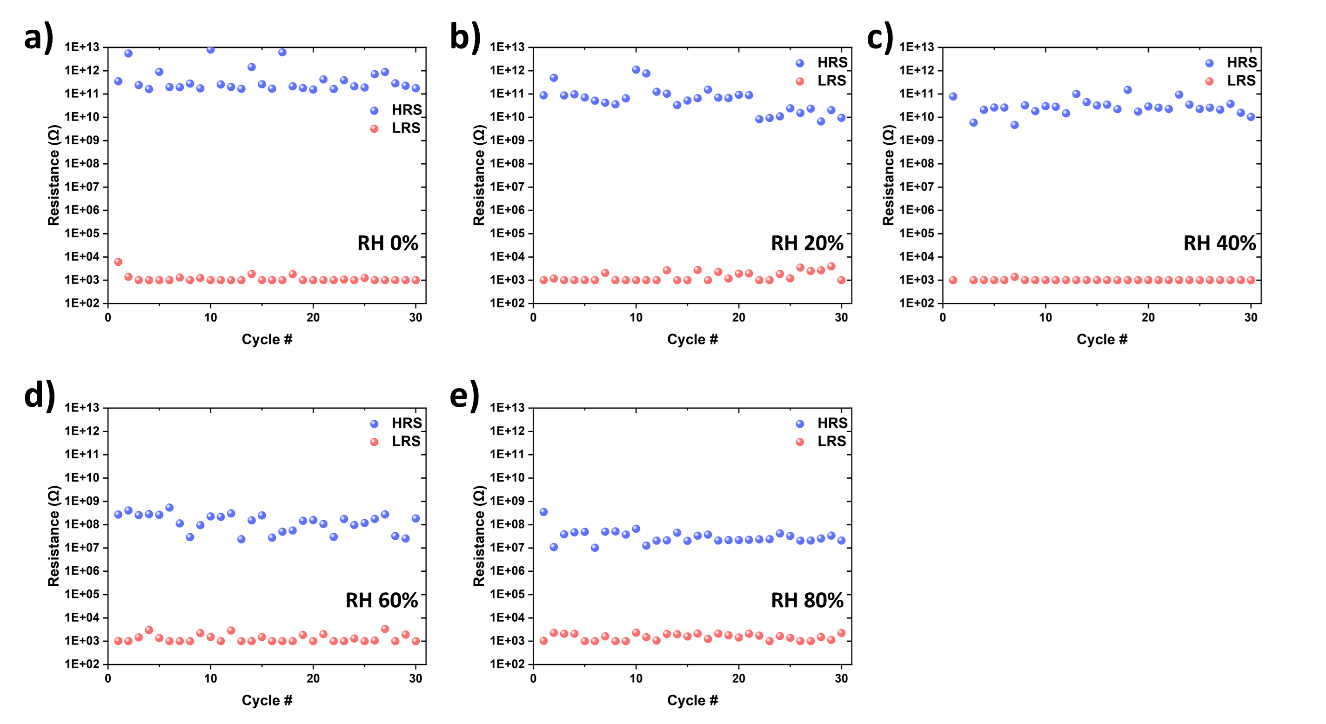
**

Figure S6. Endurance data of the Ag/Y7C/Pt memristor measured over 30 I-V cycles (0 → +10 V → 0 V → -5 V → 0 V) at five different relative humidity levels (0%, 20%, 40%, 60%, and 80% RH).

**
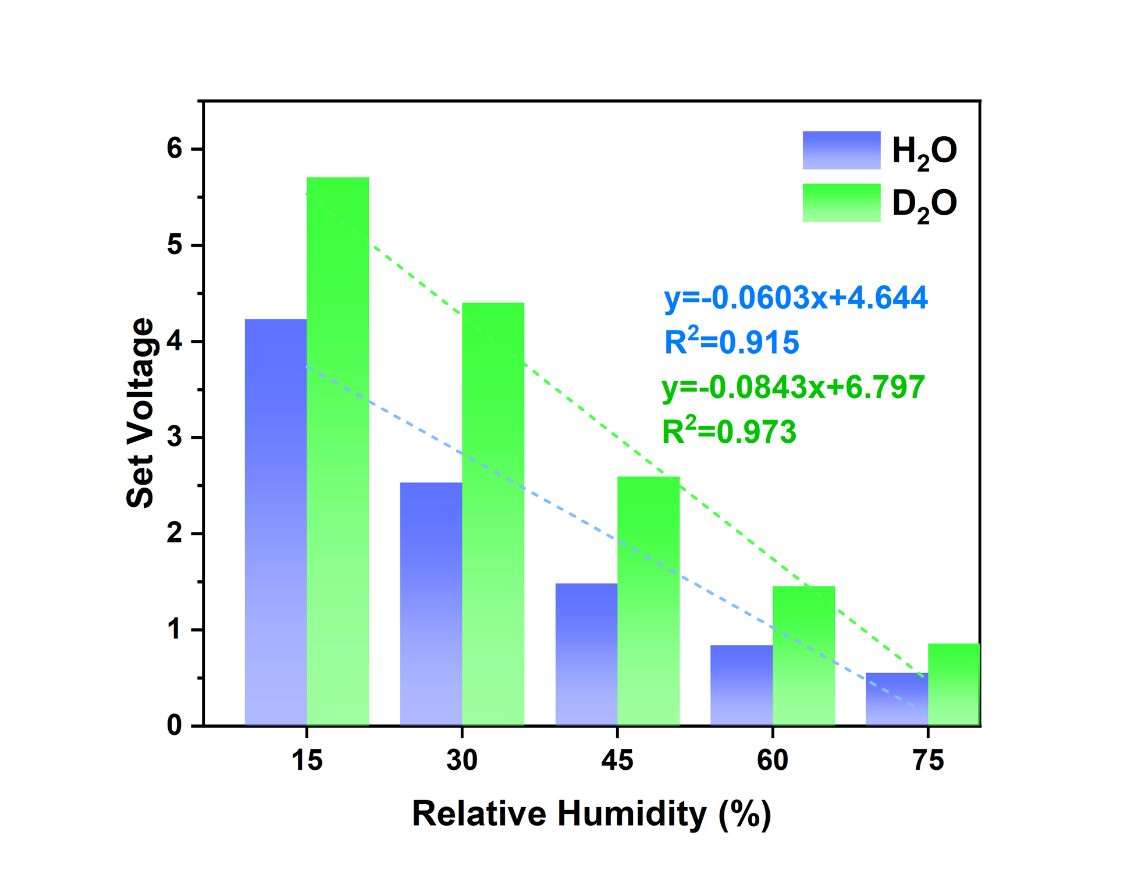
**

**Figure S7**. The set voltages of the Y7C peptide memristor as a function of the RH of H_2_O (blue) and D_2_O (green). Dotted lines indicate the linear fitting of the experimental results performed using the Origin program.

**
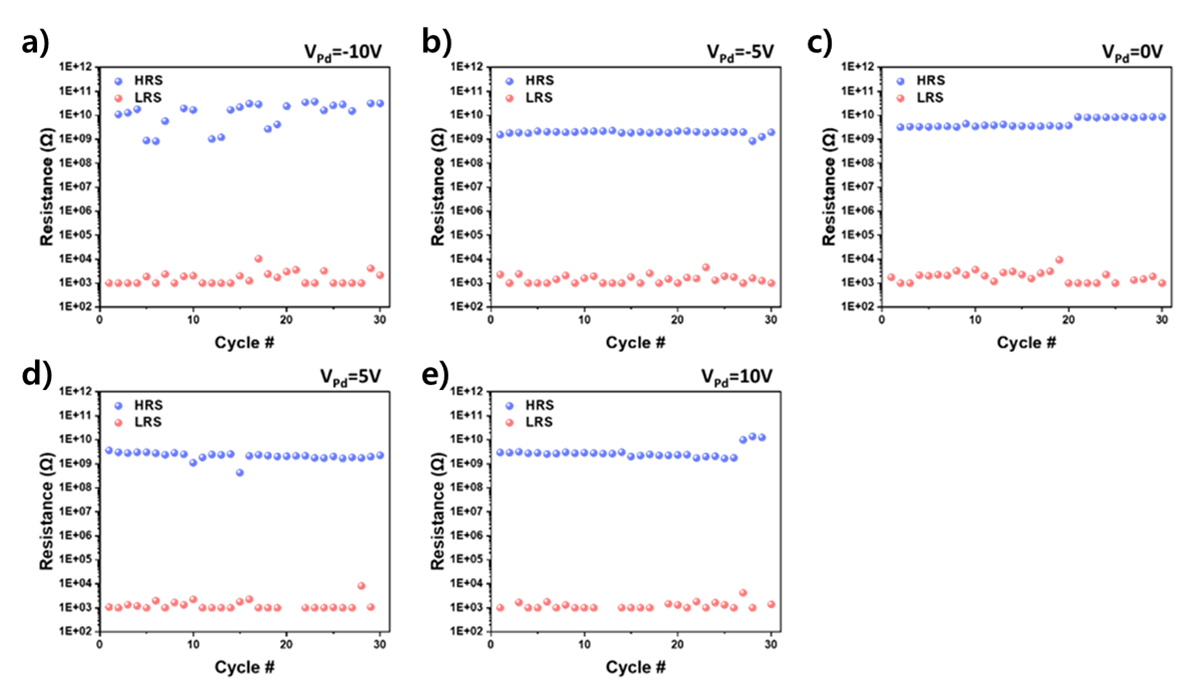
**

Figure S8. Endurance data of the Ag/Y7C/Pt memristor with adjacent Pd electrode (in 100$\boldsymbol{\mu}$m gap) measured over 30 I-V cycles (0 → +10 V → 0 V → -5 V → 0 V) with applying five voltages to Pd electrode (V_Pd_=-10 V, -5 V, 0 V, 5 V, and 10 V).

**
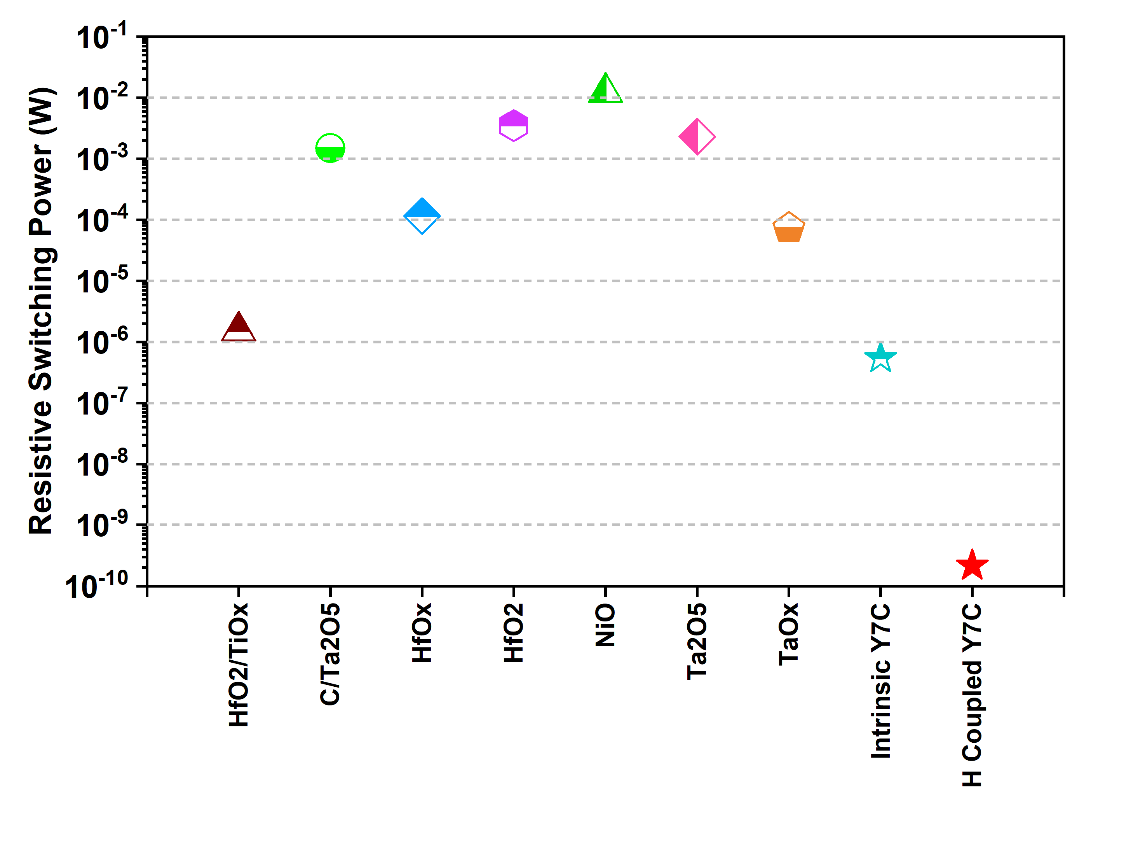
**

**Figure S9**. Resistive switching power of previously reported memristor devices based on inorganic switching media^[1–7]^.


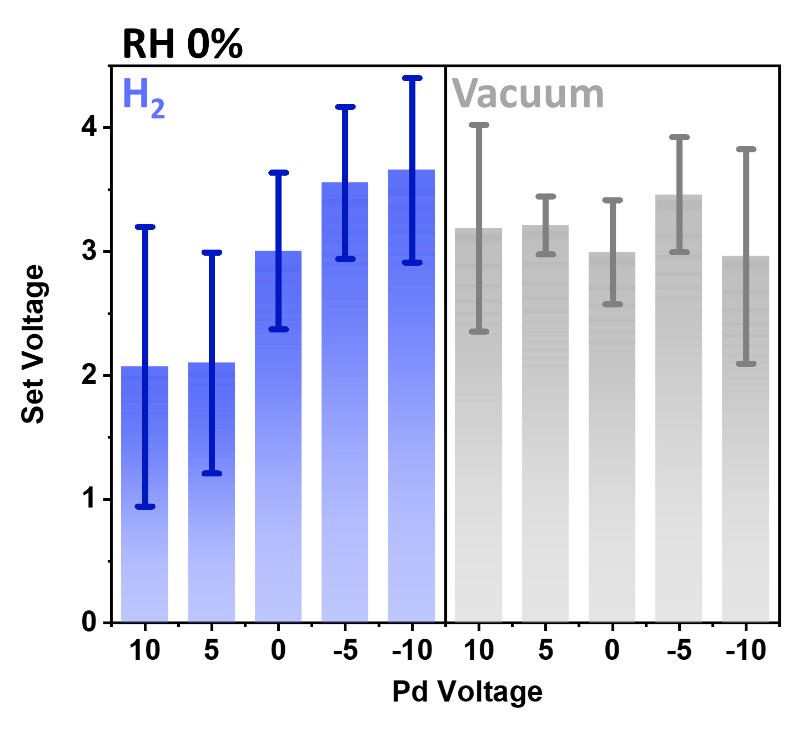


Figure S10. The set voltage distribution of the Pd-assisted Y7C memristor device in (blue) RH 0% hydrogen atmosphere, and (grey) vacuum. Each error bar represents the mean ± standard deviation (SD) calculated from a total of 20 data points for the measured set voltages. The corresponding SD% values were 54.5%, 42.5%, 21.0%, 17.3%, and 20.4% under the hydrogen atmosphere, and 26.2%, 7.3%, 14.0%, 13.4%, and 29.3% under vacuum conditions, respectively, listed in descending order of the applied V_Pd_.


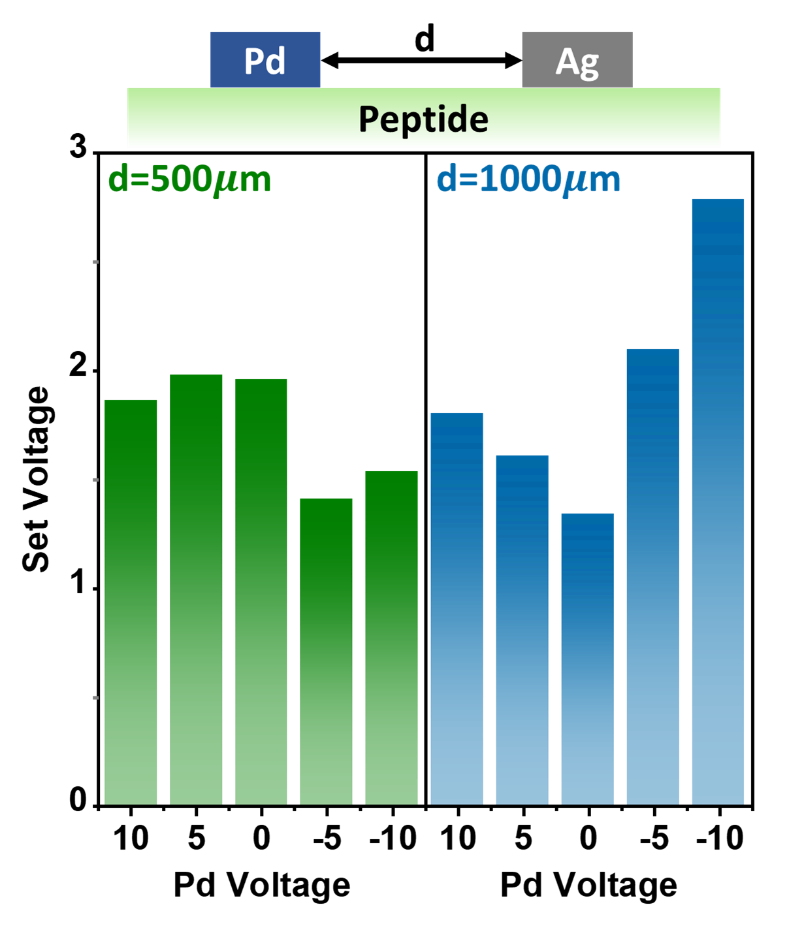


Figure S11. The set voltage distribution of the synapse-like Y7C memristor device with Ag-Pd electrode gap of (green) 500 $\boldsymbol{\mu}$m, and (blue) 1000 $\boldsymbol{\mu}$m at RH 45%, while initial devices have 100$\boldsymbol{\mu}$m gap. Each data point corresponding to a specific applied Pd voltage represents the average of 20 individual set voltage measurements.

**
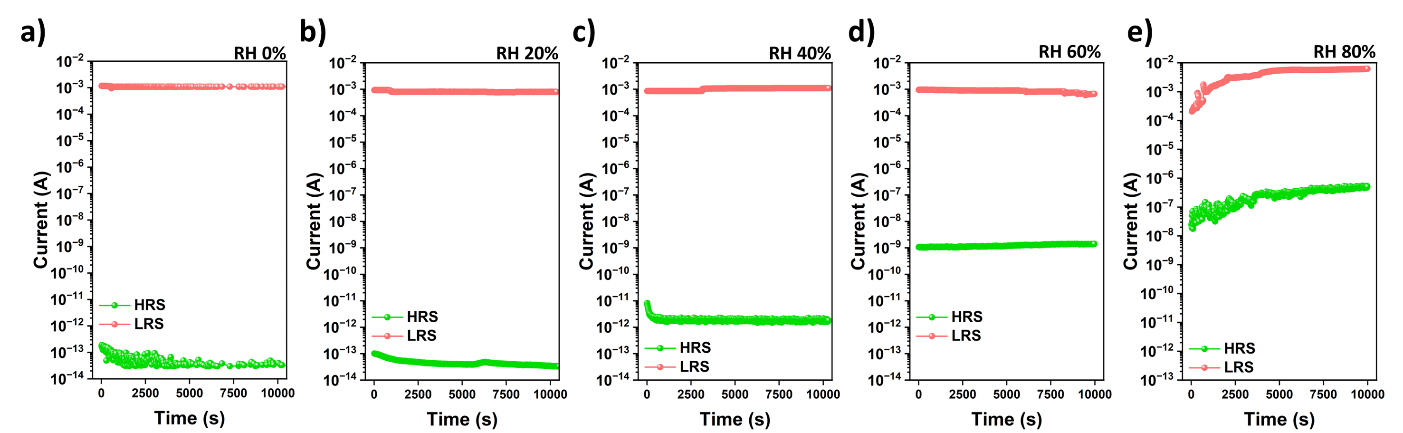
**

Figure S12. Retention data of the Ag/Y7C/Pt memristor measured over 10^4^ s at five different relative humidity levels (0%, 20%, 40%, 60%, and 80% RH).

**
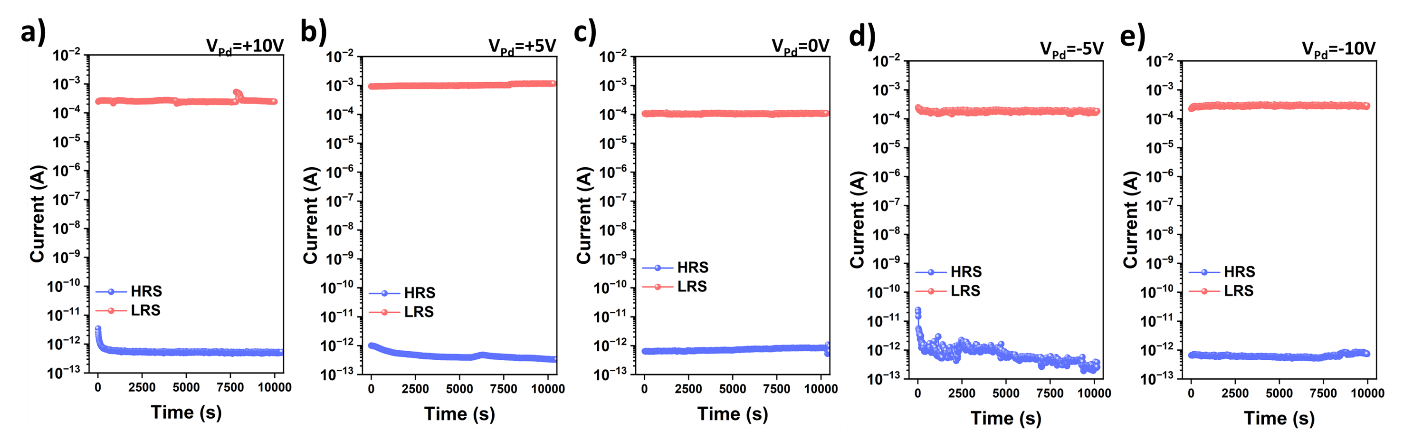
**

Figure S13. Retention data of the Ag/Y7C/Pt memristor with adjacent Pd electrode (in 100$\boldsymbol{\mu}$m gap) measured over 10^4^ s with applying five voltages to Pd electrode (V_Pd_=-10 V, -5 V, 0 V, 5 V, and 10 V).

**References**

[1] X. Ding, Y. Feng, P. Huang, L. Liu, J. Kang, *Nanoscale Res Lett* 2019, *14*, 157.

[2] H. Li, K.-S. Li, C.-H. Lin, J.-L. Hsu, W.-C. Chiu, M.-C. Chen, T.-T. Wu, J. Sohn, S. B. Eryilmaz, J.-M. Shieh, in *2016 IEEE Symposium on VLSI Technology*, IEEE, 2016, pp. 1–2.

[3] A. Schönhals, C. M. M. Rosário, S. Hoffmann‐Eifert, R. Waser, S. Menzel, D. J. Wouters, *Adv Electron Mater* 2018, *4*, 1870011.

[4] S. Gao, G. Liu, Q. Chen, W. Xue, H. Yang, J. Shang, B. Chen, F. Zeng, C. Song, F. Pan, *ACS Appl Mater Interfaces* 2018, *10*, 6453.

[5] Y. Ahn, H. W. Shin, T. H. Lee, W.-H. Kim, J. Y. Son, *Nanoscale* 2018, *10*, 13443.

[6] S. Sonde, B. Chakrabarti, Y. Liu, K. Sasikumar, J. Lin, L. Stan, R. Divan, L. E. Ocola, D. Rosenmann, P. Choudhury, *Nanoscale* 2018, *10*, 9441.

[7] J. Meng, J. M. Goodwill, E. Strelcov, K. Bao, J. J. McClelland, M. Skowronski, *ACS Appl Electron Mater* 2023, *5*, 2414.
